# Ectopic expression of transcription factor BATF3 induces B-cell lymphomas in a murine B-cell transplantation model

**DOI:** 10.18632/oncotarget.24639

**Published:** 2018-03-23

**Authors:** Christian Weiser, Mina V. Petkova, Benjamin Rengstl, Claudia Döring, Dorothee von Laer, Sylvia Hartmann, Ralf Küppers, Martin-Leo Hansmann, Sebastian Newrzela

**Affiliations:** ^1^ Dr. Senckenberg Institute of Pathology, Goethe-University of Frankfurt, Medical School, Frankfurt am Main, Germany; ^2^ Experimental and Clinical Research Center (ECRC), Medical Faculty of the Charité and Max Delbrück Center for Molecular Medicine, Berlin, Germany; ^3^ Division of Virology, Department of Hygiene, Microbiology, Social Medicine Medical University IBK, Innsbruck, Austria; ^4^ Institute of Cell Biology (Cancer Research), University of Duisburg-Essen, Medical School, Essen, Germany; ^5^ German Cancer Consortium (DKTK), Heidelberg, Germany

**Keywords:** BATF3, B-cell immunology, B-cell lymphoma, germinal center, oncogene

## Abstract

The mechanisms involved in malignant transformation of mature B and T lymphocytes are still poorly understood. In a previous study, we compared gene expression profiles of the tumor cells of Hodgkin lymphoma (HL) and anaplastic large cell lymphoma (ALCL) to their normal cellular counterparts and found the basic leucine zipper protein ATF-like 3 (BATF3) to be significantly upregulated in the tumor cells of both entities. To assess the oncogenic potential of BATF3 in lymphomagenesis and to dissect the molecular interactions of BATF3 in lymphoma cells, we retrovirally transduced murine mature T and B cells with a BATF3-encoding viral vector and transplanted each population into Rag1-deficient recipients. Intriguingly, BATF3-expressing B lymphocytes readily induced B-cell lymphomas after characteristic latencies, whereas T-cell transplanted animals remained healthy throughout the observation time. Further analyses revealed a germinal center B-cell-like phenotype of most BATF3-initiated lymphomas. In a multiple myeloma cell line, BATF3 inhibited BLIMP1 expression, potentially illuminating an oncogenic action of BATF3 in B-cell lymphomagenesis. In conclusion, BATF3 overexpression induces malignant transformation of mature B cells and might serve as a potential target in B-cell lymphoma treatment.

## INTRODUCTION

Lymphomas are a diverse group of cancers derived from B or T lymphocytes, and rarely from natural killer cells. The development of B- and T-cell lymphomas is closely linked to lymphocyte-specific genetic alterations [1]. Especially germinal center (GC) B cells are at increased risk to acquire oncogenic lesions, as these cells are exposed to extensive DNA-remodeling, i.e. somatic hypermutation and class-switch recombination [2], and are intrinsically highly proliferative [3].

Besides lesions that induce proliferation or prevent apoptosis, another mechanism of B-cell transformation involves a block in the differentiation of GC B cells into resting memory B or plasma cells [4]. During plasma-cell formation, the master regulator B lymphocyte-induced maturation protein-1 (BLIMP1) causes dramatic changes in gene expression of GC B cells [5]. The heterodimeric transcription factor complex activator protein 1 (AP-1) activates BLIMP1 expression [6]. The AP-1 factors activating BLIMP1 consist of a variety of dimers composed of members of the FOS, JUN and CREB/ATF families of proteins [7]. On the other hand, the nuclear transcriptional regulator B-cell lymphoma 6 (BCL6) plays a pivotal role in GC formation and represses BLIMP1 expression by forming inhibitory AP-1 complexes with JUN [6]. Therefore, deregulated BCL6 expression inhibits plasma-cell differentiation and can lead to lymphomagenesis [8].

The basic leucine zipper protein ATF-like 3 (BATF3) is another member of the AP-1 family and favors heterodimerization with other family members like JUN [9]. BATF3 was identified as a 21-kDa small nuclear factor isolated from T cells (p21SNFT). Several studies have analyzed the functional dimerization of BATF3 with JUN and provided evidence for its negative regulation of AP-1 mediated transcription [10, 11]. BATF3 represses AP-1 mediated IL-2 expression in Jurkat cells by binding to JUN and competitively excluding FOS from the AP-1 complex [11]. Furthermore, due to overexpression of BATF3 in hepatocellular carcinoma cells, JUN and BATF3 interact at the matrix metalloproteinase-1 (MMP-1) promoter element, resulting in repression of MMP-1 transcription [10]. However, BATF3 is also able to interact with non-AP-1 factors like IRF4 and IRF8, forming activating complexes to support lineage-specific activities [12].

In order to investigate the resemblance between tumor cells in classical Hodgkin lymphoma (HL) and their corresponding normal B-cell counterpart, we compared gene expression profiles (GEP) of isolated Hodgkin and Reed/Sternberg (HRS) tumor cells of HL, cases of diffuse large B-cell lymphoma (DLBCL), several HL cell lines and human GC B cells. These analyses revealed strong and specific overexpression of BATF3 in HL cell lines as well as primary HRS cells in comparision to normal GC B cells and DLBCL [13, 14]. In another study, BATF3 was found to be highly upregulated in the T-cell malignancy anaplastic large cell lymphoma (ALCL) [15]. The observations instigated further investigation of a potential role of BATF3 in lymphomagenesis. Therefore, we established a tumor mouse model to illuminate the transformation potency of BATF3 in mature lymphocytes.

## RESULTS

### BATF3 is highly expressed in B-cell lymphomas and ALCL

**Figure 1:**
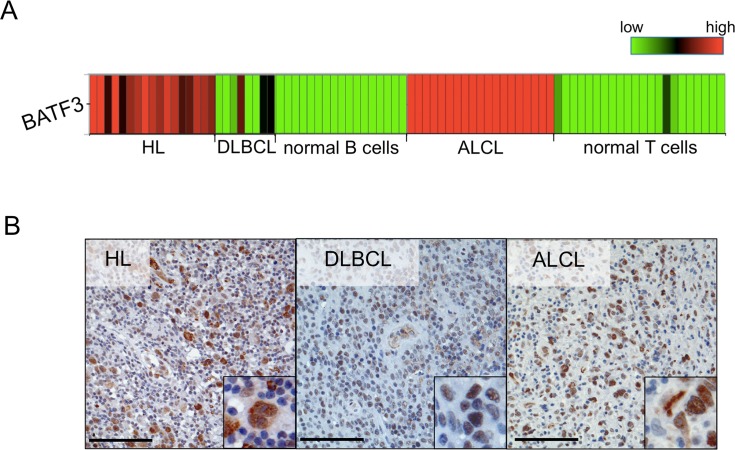
BATF3 is aberrantly expressed in several lymphoma entities **(A)** Heatmap highlighting the upregulated gene expression of BATF3 in Hodgkin lymphoma (HL), anaplastic large cell lymphoma (ALCL) and some diffuse large B-cell lymphoma (DLBCL) cases, when compared to the expression in normal human B (mature, germinal center and plasma cells) and T cells (mature CD4^+^ and CD8^+^ cells). BATF3 gene expression data were extracted from Brune et al. [16] and Eckerle et al. [15]. **(B)** Representative immunohistochemistry of BATF3 in HL, DLBCL and ALCL, displaying nuclear BATF3 expression. Bars 100 μm (200 x, insert 400 x).

In previous studies, we performed GEP of HL, DLBCL, ALCL, and normal B and T cells [14–16]. When compared to their normal cellular counterparts, we identified BATF3 as highly and consistently upregulated in the tumor cells of HL and ALCL, and also upregulated in some DLBCL (Figure 1A). To confirm this overexpression of BATF3 at protein level, immunohistochemistry of B- and T-cell lymphomas was carried out. We investigated 12 primary HL, 21 DLBCL and 18 ALCL (Table 1). A strong nuclear expression of BATF3 in the tumor cells was confirmed in all cases of HL, in 11 of 21 DLBCL and 14 of 18 ALCL (Figure 1B and Table 1). In our prior analysis of ALCL 41 of 41 ALCL showed strong expression of BATF3 [15]. Reactive tonsils were evaluated as a control and revealed a nuclear BATF3-expression only in some GC B cells (not shown, see also Discussion).

**Table 1:** Immunohistochemical analysis of BATF3 expression

Entity	BATF3-positive cases/total cases (%)
DLBCL	11/21 (52)
HL	12/12 (100)
ALCL	14/18 (78)

*BATF3* mutation analysis was performed for all ALCL and DLBCL primary cases and three HL cell lines (L428, L-1236, KM-H2). In none of the samples we identified any mutations in the coding sequence of *BATF3* (data not shown).

### Ectopic expression of human BATF3 provokes B-cell lymphoma in a murine transplantation model

**Figure 2:**
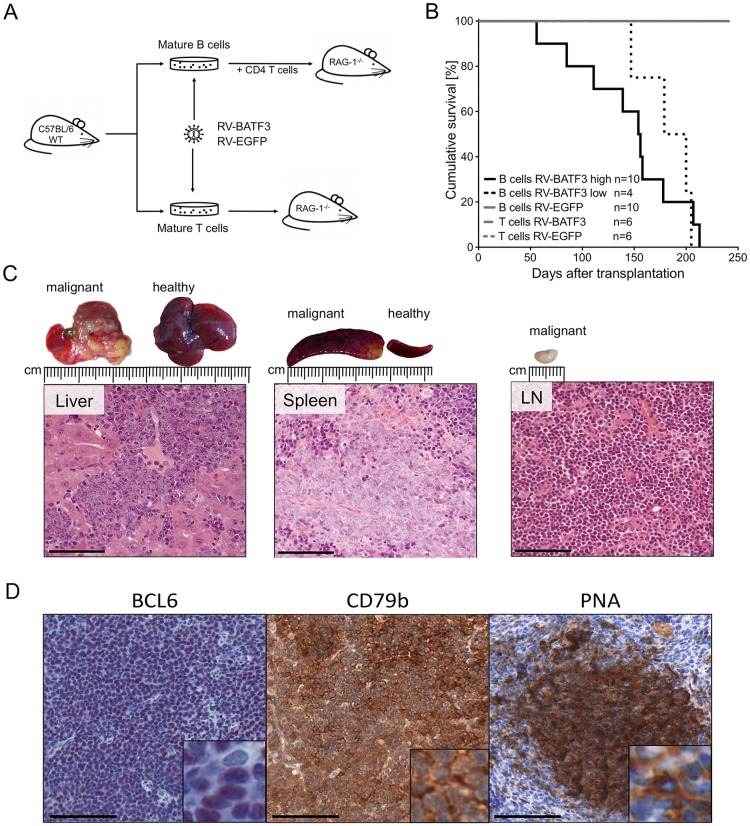
Overexpression of BATF3 in mature B cells results in lymphomagenesis **(A)** Experimental design and transplantation model. Wild type (WT) C57BL/6 mice were used as donors for mature B and T cells. Isolated cells were stimulated in cell culture, retrovirally transduced and transplanted into Rag1-deficient recipient mice. B-cells were co-transplanted with freshly isolated, TCR quasi-monoclonal, CD4^+^ T cells from OT-II mice. Mice were monitored by blood sampling and monitored for lymphoma development. **(B)** Survival of T- and B-cell transplanted recipients. Cohorts transplanted with BATF3-expressing B cells (black solid and dotted lines) developed lymphomas after different latencies. Animals that received highly BATF3-transduced B cells (black solid line) succumbed earlier to lymphoma than recipients of low BATF3-expressing B cells (black dotted line). One of the mice transplanted with cells transduced with high levels of BATF3 vector died of lymphoma, but could not be included in the detailed characterization of the lymphomas because of insufficient quality of the necrotic tissue. Recipients of BATF3-expressing T cells and EGFP-control cells did not show any signs of malignancy during the observation period (gray lines). **(C)** Representative histologies of a BATF3-induced B-cell lymphoma. Necropsy of diseased animals revealed massive infiltration of tumor cells into the liver, spleen and lymph nodes. Hematoxilin and eosin stained tumors demonstrated blastoid infiltrates and a destruction of the normal, organ-specific architecture. Bars represent 100 μm. **(D)** Representative immunohistochemical stainings of BATF3-induced tumors. Sections of tumor-infiltrated spleen of BATF3-induced lymphomas were stained for the B-cell marker CD79b and the GC B-cell markers BCL6 and PNA. Bars represent 100 μm.

**Figure 3:**
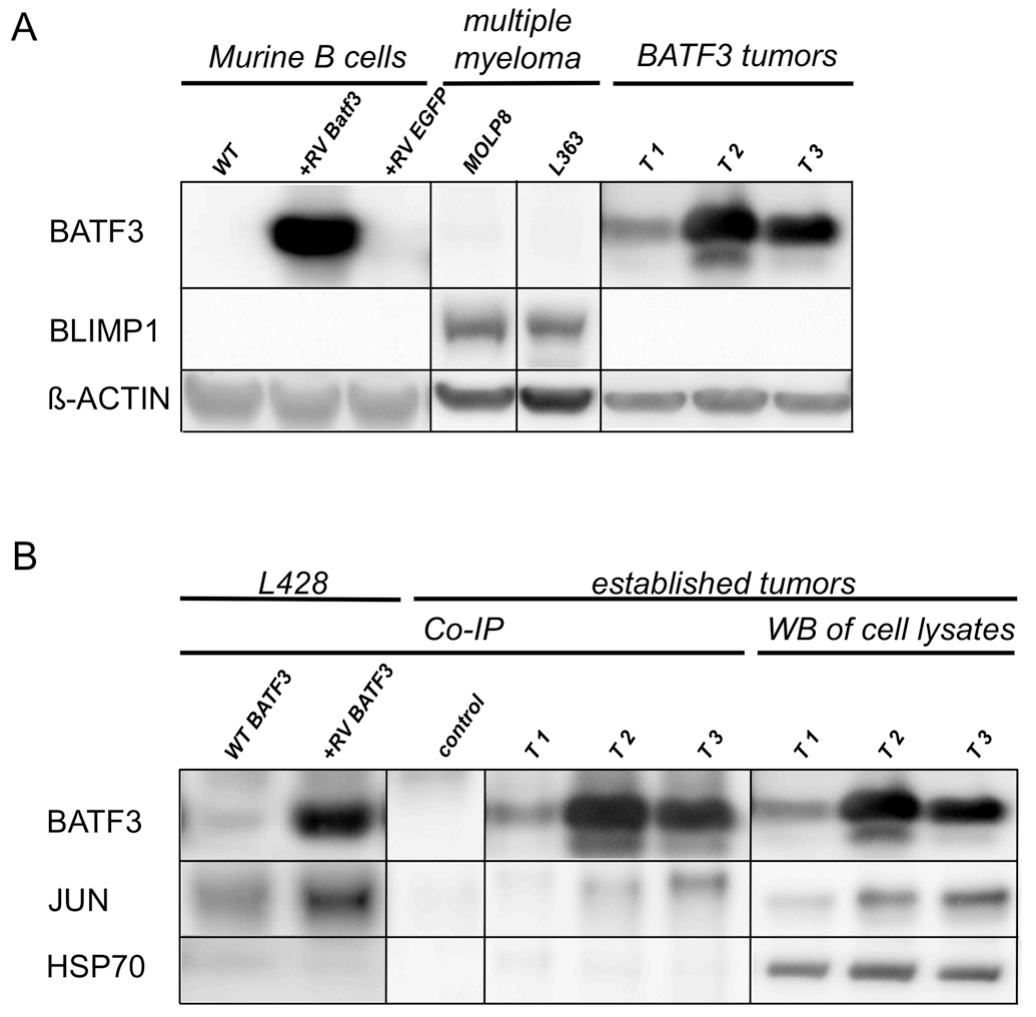
BATF3 interacts with JUN in BLIMP1-negative, BATF3-induced tumor cell lines **(A)** Western blot analysis of LPS-stimulated, freshly transduced murine B-cells and established murine cell lines from BATF3-induced tumors (lanes T1-3) for the expression of BATF3, and BLIMP1. RV indicates retroviral transduction with BATF3 or EGFP as a control. Multiple myeloma cell lines MOLP8 and L363 were used as positive control for BLIMP1 expression. **(B)** Co-IP of BATF3 and JUN with HL cell line L428 and BATF3-induced murine B-cell tumor cell lines. L428 cells showed an endogenous expression of BATF3, therefore JUN was immunoprecipitated in wild type (WT) L428 cells (WT BATF3 lane). As expected, retroviral expression of BATF3 in L428 cells even increased the amount of retained JUN protein after Co-IP (+RV-BATF3 lane). Most interestingly, interaction of BATF3 and JUN was demonstrated in BATF3-induced tumors (lanes T1-3). Western blot of the corresponding whole lysates and after Co-IP without specific antibodies served as controls. HSP70 was used as negative control for Co-IP experiments. All Co-IP experiments were performed with a BATF3-specific antibody.

To investigate a potential oncogenic function of upregulated BATF3 expression in lymphocytes, we isolated mature T and B cells from spleen and lymph nodes of wild type C57BL/6 mice. After *in vitro* stimulation of the isolated lymphocytes, we retrovirally transduced the cells with the human *BATF3* gene, which has 80% aminoacid sequence identity with the murine counterpart. The *BATF3* encoding gammaretroviral vector coexpressed enhanced green fluorescence protein (EGFP) as a marker gene via an internal ribosomal entry site (IRES) to enable detection of transduced cells (Supplementary Figure 1A). As a control, T and B lymphocytes were transduced with the marker EGFP only. B cells were the primary target of our investigations; therefore, we prepared a high and a low copy batch of cells for transplantation (Supplementary Figure 1B). Before transplantation the phenotype of the modified B cells was determined (Supplementary Table 1). Subsequently, transgene-expressing B and T cells were separately transplanted into lymphopenic Rag1-deficient recipients (Figure 2A). To enable a better engraftment, mature B cells were co-transplanted with supporting CD4^+^, T-cell receptor (TCR)-transgenic OT-II T cells. Intriguingly, after transplantation of *BATF3*-transduced B cells, all 14 transplanted animals developed malignancies with a latency of 56-213 days (Figure 2B). Apparently, the gene expression intensity influenced the development of the malignancies, as recipients of highly *BATF3*-transduced B cells succumbed earlier to lymphoma. Necropsy of diseased animals and histological analyses of isolated organs demonstrated massive liver infiltrates, splenomegaly and lymphadenopathy (Figure 2C). Moreover, Western blot analysis revealed high expression of BATF3 in the lymphomas, with a considerable variation between cases (Figure 3, Supplementary Figure 2A). To study the clonality of the lymphoproliferations, we used a ligation mediated-PCR approach [17, 18]. As the retroviruses used for transduction of mature B cells integrate randomly into the murine genome, amplification by ligation-mediated PCR of a DNA fragment with one particular restriction site within the vector and the next site in the neigboring genomic DNA can serve as an elegent marker for clonality of the lymphoproliferations, because nearly all integration events will generate fragments of distinct sizes. This analysis demonstrated a mono- to oligoclonal character of the 13 malignancies analysed (Supplementary Figure 2B; the necropsy material from one mouse was of insufficient quality for molecular analysis). As clones might occasionally have two or more viral integrations, even some samples with 2-3 bands may be monoclonal. In one of the transplanted mice, monoclonality of the B cells was further supported by expression of λ light chains by the vast majority of B cells, because only 5% of normal polyclonal murine B cells express this type of light chain (Table 2). Flow cytometric and immunohistological examination identified a GC B-cell-like phenotype of the lymphoma cells of most lymphomas (Table 2 and Figure 2D). Seven of the 13 lymphomas expressed Bcl6, the master regulator of the GC B cell program, and 11/13 were double-positive for the GC B-cell markers GL7 and Fas. Furthermore, the lectin peanut agglutinin (PNA), a further characteristic feature of GC B cells, was expressed by 7/13 lymphomas (Figure 2D). Histologies of tumors demonstrated blastoid infiltrates and effacement of the normal, organ-specific architecture, consistent with aggressive B-cell lymphoma of diffuse large cell type.

**Table 2:** Phenotype of BATF3-induced lymphomas

	CD19/B220 double positive	GL7/Fas double positive	CXCR4	CXCR5	IgM	IgD	IgG	Igλ	Igκ	CD86	CD83	Bcl6	PNA	CD79b	WBC
BATF3-induced lymphomas n=13	10/13	11/13	8/13	10/13	4/13	1/13	7/13	1/13	9/13	11/13	8/13	7/13	7/13	13/13	6/13

Only tumors presenting more than 70% positive cells, as determined by flow cytometry, were set as positive.

BCL6, PNA and CD79b were determined by immunohistochemistry. WBC, white blood cell count [>15000/μl].

### BATF3-induced B-cell tumors lack BLIMP1 expression

To unravel mechanisms involved in the lymphomagenesis of BATF3-triggered B-cell malignancies, we compared the expression of the AP-1 target gene BLIMP1 in freshly BATF3-transduced murine B cells and three B-cell lines established from our BATF3-induced lymphomas. As expected from the GC B-cell phenotype of the lymphomas, we did not detect any expression of this plasma cell master regulator in the tumors and/or the freshly stimulated and transduced B cells. Two multiple myeloma (MM) cell lines served as positive control for upregulated BLIMP1 expression (Figure 3A). This prompted us to investigate whether BATF3 dimerizes with JUN in B cells and thereby possibly represses AP-1 function and BLIMP1 expression. To test this hypothesis, we performed co-immunoprecipitation (Co-IP) experiments with the BATF3-induced lymphoma cell lines. Initially and as a control, we transduced HL-cell line L428 with BATF3 and tested the cells for BATF3-JUN dimers by Co-IP analyses. As L428 cells already show endogenous BATF3-expression, we were able to capture JUN in untreated L428 cells after Co-IP with BATF3. This result was enhanced when we ectopically expressed BATF3 in this cell line. Most intriguingly, the binding of JUN to BATF3 was also detectable in the cell lines of the BATF3-induced lymphomas (Figure 3B).

### BATF3-induced downregulation of BLIMP1 triggers cell death in myeloma cells

As a next step, we wanted to functionally analyze the potency of BATF3-mediated inhibition of BLIMP1 expression in malignant B cells. Therefore, we analyzed BLIMP1-expressing MM cell lines, although in these cells BLIMP1 has an oncogenic and not a tumor-suppressive function as in mature B-cell lymphomas. We first tested BLIMP1-dependent survival of MM cell lines MOLP8 and L363. As expected and already reported [19], both cell lines demonstrated significantly impaired cell growth after targeting BLIMP1 expression by lentivirally-mediated transfer of BLIMP1-specific shRNAs (Supplementary Figure 3).

**Figure 4:**
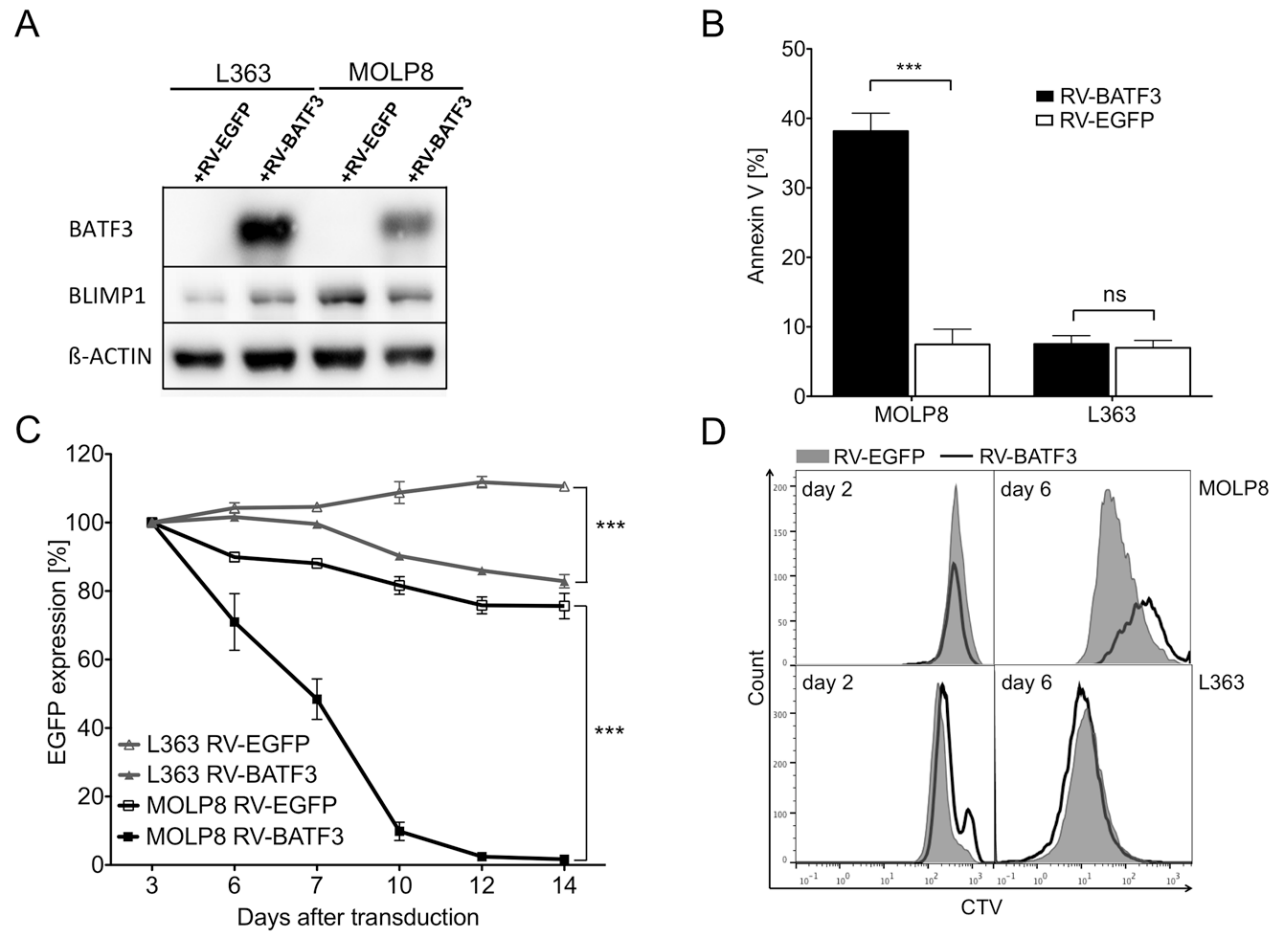
BATF3-overexpression leads to downregulation of BLIMP1 and apoptosis in multiple myeloma (MM) cell line MOLP8 **(A)** Expression of BLIMP1 and BATF3 in MM cell lines MOLP8 and L363 after retroviral transduction with BATF3 (+RV-BATF3) or EGFP (+RV-EGFP) as a control. Ectopic BATF3-expression induced a downregulation of BLIMP1 in MOLP8 cells, whereas BLIMP1 was found to be upregulated in L363 cells after BATF3-transduction. **(B)** BATF3-induced apoptotic cell death in MM cell line MOLP8. Seven days after transduction with BATF3, MOLP8 cells showed an increased fraction of apoptotic cells (left black bar) when compared to control-gene transduced cells (left open bar). BATF3-overexpression in L363 cells did not lead to increased apoptosis (right black and open bars). **(C)** BATF3-expression dramatically impaired cell growth of MM cell line MOLP8 (black line with solid square). On the other hand, control-gene modified MOLP8 cells (black line with open square) and transduced L363 cells (grey line with solid or open triangles) did not show reduced expansion. **(D)** Proliferation of MM cell lines MOLP8 and L363 after transduction with BATF3 or control gene EGFP. Directly after transduction, both cell lines were labeled with the cell-division tracker CellTrace Violet (CTV) and were analyzed by flow cytometry on days 2 and 7. Subsequently, flow cytometric analysis on day 7 revealed that BATF3-expressing MOLP8 cells (solid line) compared to EGFP-modified control cells (grey area) showed a reduced proliferation. L363 cells were not affected in their proliferation capacity after BATF3-overexpression. Error bars represent standard deviation. Statistical significance was established with a paired *t* test. All experiments were performed in triplicates. ^***^, P < 0.0001, ns, not significant

Next, we transduced the BLIMP1-expressing cell lines with BATF3, to enforce inhibition of AP-1 and thereby BLIMP1 expression. As expected, in MOLP8 cells BATF3-overexpression resulted in downregulation of BLIMP1. Surprisingly, ectopic expression of BATF3 led to an unaffected BLIMP1 levels in L363 cells (Figure 4A). Consequently, BATF3 expression had no impact on the survival of L363 cells. In contrast, BATF3-mediated inhibition of BLIMP1 dramatically impaired growth of MM cell line MOLP8 (Figure 4B), by induction of apoptosis and inhibition of cell proliferation, thus validating that BATF3 can indeed suppress BLIMP1 expression (Figure 4C and 4D).

## DISCUSSION

In a study of differential gene expression of HL cell lines, we observed increased BATF3-expression [13]. This finding was validated in a larger Affymetrix GEP analysis of HL cell lines and primary HRS cells in comparison to other B-cell lymphomas, and the main subsets of normal mature B cells [14, 16]. Importantly, high BATF3 expression was specifically seen in HRS cells. In a similar GEP study of isolated tumor cells of ALCL in comparison to eight subsets of normal mature T and natural killer cells, high BATF3 expression was specifically seen in ALCL tumor cells [15]. We demonstrated a strong expression of BATF3 on protein level in HL, ALCL, and a fraction of DLBCL. These findings are in line with two recent studies which also revealed BATF3 protein expression in 70% of classical HL, in 30% of CD30^−^ DLBCL, in over 60% of CD30^+^ DLBCL, and in about 90% of primary mediastinal B cell lymphomas [20]. Notably, among normal B cells, BATF3 is only expressed by very few GC and extrafollicular B cells that also express CD30 [20, 21]. Altogether, these analyses provided compelling support for the notion that BATF3 might play a pivotal role in several types of B- and T-cell tumors. We did not detect any genetic alterations in the coding sequence of BATF3 in the human lymphomas that might explain the strong BATF3 expression. However, we and others recently showed that *BATF3* is a direct target of STAT factors and of the PI3K/AKT pathway [20, 21]. As both of these are constitutively active in HRS cells of classical HL, in primary mediastinal B cell lymphoma, and ALCL [22–25], STAT and PI3K/AKT activities are main contributors of BATF3 expression in these lymphomas, as functionaly validated in HL cell lines [20, 21].

To investigate the potential tumor-initiating capacity of BATF3 in lymphomagenesis of B and T cells, we retrovirally transduced murine mature T and B cells with human *BATF3* and transplanted the cells into immunocompromised recipients. T-cell transplanted animals did not show any sign of malignancy during the whole observation time of more than 250 days. Moreover, we overexpressed BATF3 in human T-cell lines, but did not observe any change in proliferation or enhanced cell survival (data not shown). Most likely, BATF3 is not a strong oncogene in T cells and needs further synergistic genetic events in order to induce transformation of mature T cells. Nevertheless, we recently showed that downregulation of BATF3 in ALCL cell lines is toxic for the cells, supporting oncogenic features of BATF3 also in this T-cell malignancy [21]. In human BATF3-positive ALCL, the NPM-ALK translocation may serve as a critical cooperating genetic lesion that is missing in the mouse model.

Strikingly, BATF3-expressing B cells readily induced B-cell lymphomas in all transplanted mice. Our murine B-cell transplantation model bears certain experimental limitations. First of all, Rag1-deficient recipients do not feature a normal adaptive immune system, and the co-transplanted CD4^+^ T cells expressed an irrelevant transgenic, ovalbumin-specific TCR, so that we can exclude a T-cell dependent GC formation in our mouse model. Moreover, lymphoma-affected tissues of diseased animals featured a low number of accompanying T cells (data not shown). Unexpectedly, the B-cell tumors that developed in the transplanted mice mostly had a GC B-cell phenotype, with expression of the GC B-cell markers Fas and GL7 by nearly all lymphomas, and Bcl6 and PNA each by about half of the cases (Table 2). This might be explained by thymus-independent mechanisms and the stimulation via LPS in our experimental setting [26], or a partial GC B-cell program-inducing effect of BATF3. As the recipient Rag1-deficient mice lack polyclonal T cells and a normal architecture in lymph nodes and spleen, the lymphoproliferations can not be clearly linked to a specific type of human lymphoma in terms of the histological picture. The infiltrates have mostly a diffuse appearance, but for the reason just mentioned, we refrain from typing them as DLBCL.

Normally, almost all GC B cells display a BCL6^+^ and BLIMP1^−^ phenotype, only a minority of cells upregulate BLIMP1 to enable plasma-cell differentiation, which causes BCL6 downregulation [10, 11, 27]. However, modification of BCL6 expression can cause disturbances of BLIMP1-mediated differentiation cascades and subsequently lead to lymphomagenesis [28, 29]. Here, we might observe that upregulated BATF3, with functional repression of the AP-1 complex results in an impaired BLIMP1 expression and in a differentiation block-induced GC B-cell transformation. Indeed, the findings of our Co-IP studies hint at an interaction between BATF3 and JUN (Figure 3B), which might be an explanation for the missing BLIMP1 expression in our B-cell lymphomas. Moreover, in the functional experiments, we were able to show that BATF3 is capable to induce BLIMP1 downregulation and apoptosis in MM cells (Figure 4B, 4C). This is in line with a recent report that BATF3 downregulates BLIMP1 expression in the HL cell line L428 [20]. As the B cells lacked BLIMP1 expression prior to transduction with BATF3-encoding retroviruses, it remains presently unclear whether a prevention of BLIMP1 upregulation is the main pathogenetic effect of BATF3 in this mouse lymphoma model. Indeed, we recently showed that in HL and ALCL, *MYC* is a direct target of BATF3, so that the promotion of constitutive expression of this proto-oncogene is presumably a main pathogenic function of BATF3 in these lymphomas [21].

In summary, we established BATF3 as a potent oncogene in murine B cells. As two recent studies restricted to human HL cell lines revealed toxic effects of downregulating high BATF3 expression in these cells [20, 21], our study provides essential data showing an oncogenic feature of BATF3 in mature B cells *in vivo*, and thereby strongly supports its pathogenetic role in HL and also some DLBCL.

## MATERIALS AND METHODS

### Mice

Six- to eight-week-old C57BL/6J.Ly5.1 (CD45.1^+^), OT-II and Rag1-deficient (CD45.2^+^) mice were obtained from Charles River Laboratories (Sulzfeld, Germany) and from the commercial breading facility mfd Diagnostics GmbH (Wendelsheim, Germany). Animals were maintained under specific pathogen-free (SPF)-like conditions in the animal facilities of the Georg-Speyer-Haus (Frankfurt, Germany). Cages were individually ventilated. Animals were killed after anesthesia by cervical dislocation. Diseased animals were examined for pathologic abnormalities, including histology, morphology, white blood cell counts and flow cytometry. The experiments were performed in compliance with the local animal experimentation guidelines. Animal experiments were approved by the regional council (Regierungspräsidium, Darmstadt, Germany).

### Retroviral vectors and cloning

The human *BATF3* gene was synthesized and codon optimized for mouse expression by GeneArt^®^ (Life Technologies, Darmstadt, Germany). Control vector MP91-EGFP was described elsewhere [30]. The synthesized cDNA was cloned into the gammaretroviral vector MP91-EGFP in front of an internal ribosome entry site from the encephalomyocarditis virus and the gene for EGFP.

BLIMP1-specific targeting oligonucleotides and non-specific sequences as a control (described elsewhere [19]) were used for cloning of shRNA-encoding sequences into the lentiviral expression vector LeGO-Cer, kindly provided by Kristoffer Riecken [31]. The small hairpin ribonucleic acid (shRNA) sequence with its H1 promoter was located in front of a SFFV promoter that regulates expression of cerulean as reporter gene.

### Production of retroviral vector particles

Vector supernatants were produced in DMEM GlutaMax^®^ (Life Technologies) supplemented with 10% fetal bovine serum (FBS) (Biochrom GmbH, Berlin, Germany) and 1% penicillin/streptomycin (Pen/Strep). Retroviral supernatant was produced in a split genome approach by calcium phosphate mediated transient transfection of HEK 293T human embryonic kidney producer cells. After 36 and 48 hours supernatant was collected, filtered (0.2 μm pores) and stored at 4°C. All supernatants were titrated on the embryonic murine fibroblast SC-1 cell line or HEK 293T cells.

### Cell culture conditions, retroviral transduction and transplantation

Murine B and T cells were isolated from spleen and lymph nodes (mesenteric and superficial inguinal) of C57BL/6J.Ly5.1 donor mice. After preparation of single cell suspensions untouched isolation of the two different cell types was performed with the respective isolation kits (B cells: Miltenyi Biotec GmbH, Bergisch Gladbach, Germany); T cells: Dynabeads^®^, Life Technologies). Stimulation and transduction of T cells was previously described [32]. B cells were stimulated with lipopolysaccharides (LPS) from E. coli 0111:B4 (Sigma-Aldrich, Munich, Germany) for 3 days. At day 1 after stimulation, B cells were transduced on retronectin-coated (Takara Bio Inc., Tokyo, Japan), vector supernatant-preloaded (5 ml vector supernatant per well; 1000 g, 31°C, 30 min) non-tissue culture plates. Stimulated mature B cells were kept in RPMI 1640 GlutaMax™ (Life Technologies), supplemented with 10% FCS, 1% L-glutamine, 1% Pen/Strep, 1% sodium pyruvate, 1% nonessential amino acids, and 0.1% β-mercaptoethanol (Life Technologies) throughout the entire cultivation period. Culture conditions included LPS at 10 μg/ml. Three days after isolation and two rounds of transduction, 5×10^6^ transduced mature T or B cells were injected intravenously into the tail vein of Rag1-deficient recipient mice.

Myeloma cell lines L363 and MOLP8 (DSMZ, Braunschweig, Germany) were transduced on retronectin-coated, vector supernatant-preloaded (5 ml vector supernatant per well; 1000 g, 31°C, 30 min) non-tissue culture plates. Three days after transduction Cerulean- or EGFP-expressing cells were enriched via fluorescence activated cell sorting on an S3-Cell sorter (Bio-Rad Laboratories, Munich, Germany) and cultured under standard culture conditions (37°C in a humidified atmosphere containing 5% CO_2_). Cell lines were grown in RPMI 1640 GlutaMax™ (Life Technologies), with 1% Pen/Strep. L363 cells were cultured with 10% FCS, and MOLP8 were cultured with 20% FCS.

### Establishment of BATF3-expressing tumor cell lines

Cell suspensions from primary mouse tumor material were prepared and cultivated in RPMI 1640 GlutaMax™ (Life Technologies), supplemented with 10% FBS (Biochrom), 1% L-glutamine, 1% Pen/Strep, 1% sodium pyruvate, 1% nonessential amino acids, and 0.1% β-mercaptoethanol (Life Technologies). After two weeks in culture, the surviving, non-adhering cells were isolated and further cultivated. Three B-cell lines (T1-T3) were raised from primary mouse tumors.

### Flow cytometry

Flow cytometric analyses were performed on peripheral blood and single-cell suspensions of spleen, lymph nodes, bone marrow and solid tumors. The following anti-mouse antibodies were used for staining: rat anti-mouse monoclonal antibodies (eBioscience, San Diego, CA, USA) phycoerythrin cyanine7 (PE-Cy7) conjugated anti-CD19 (1D3), PE-Cy7 conjugated anti-IgG1 (M1-14D12), PE-Cy7 conjugated anti-CD38 (90), PE-Cy7 conjugated anti-CD86 (GL1), allophycocyanin (APC)-conjugated anti-IgM (II/41), APC conjugated anti-CXCR4 (2B11), eFluor^®^450-conjugated anti-MHC-II (M5/114.152.2) phycoerythrin (PE) conjugated anti-IgG, APC conjugated anti-IgD (11-26c), (BioLegend, San Diego, CA, USA) APC conjugated anti-CD45R/B220 (RA3-6B2), APC conjugated anti-CD83 (Michel-19), allophycocyanin cyanine7 (APC-Cy7)-conjugated anti-Igκ light chain (RMK-45), (Becton Dickinson (BD) Pharmingen, Heidelberg, Germany) PE-Cy7 conjugated anti-CXCR5 (2G8), APC conjugated anti-CD138 (281-2), PE conjugated anti-GL7 (Miltenyi Biotec), VioBlue conjugated anti-CD8a (53-6.7); hamster anti-mouse monoclonal antibody (BD Horizon), BV421 conjugated anti-Fas (Jo2); goat anti-mouse polyclonal antibody (Beckman Coulter, Brea, CA, USA), PE conjugated anti-Igλ light chain. To prevent nonspecific binding of antibodies to Fc receptors, samples were incubated with mouse FcR Blocking Reagent (Miltenyi Biotec). Before staining, samples of blood, bone marrow and spleen were treated with Lysing Buffer (BD) to remove erythrocytes. Propidium iodide (Miltenyi Biotec) was used to exclude dead cells. Apoptotic cells were determined by Annexin V-APC staining (BD Pharmingen) and cell proliferation assay was performed by CellTrace Violet (Life Technologies) staining, according to the manufacturer´s protocols. All flow cytometric analyses were performed on a MACSQuant (Miltenyi Biotec) using the FlowJo 8.7 software (FLOWJO LLC, Ashland, USA).

### Western blot analyses

For Western blot analyses, 1-5 × 10^6^ cells were lysed in 1x RIPA buffer (Cell Signaling, Danvers, USA), including protease inhibitor cocktail (Roche, Basel, Switzerland). Protein concentration was determined by DC protein assay (Bio-Rad Laboratories) and 40 μg per lane and sample were loaded. Western blotting was performed according to the operating instructions of the device manufacturer. Rabbit anti-mouse/−human antibodies were used in a 1:1000 dilution: (Cell Signaling Technology, Leiden, The Netherlands), anti-Blimp1 (C14A4) (Cell Signaling Technology), anti-HSP70 (Cell Signaling Technology), anti-JUN (Genway Biotech, San Diego, USA), and mouse anti-human BATF3 antibody (3H1) (Abnova, Taipei, Taiwan) in a 1:5000 dilution and as loading control mouse anti-mouse/−human antibody anti-β-actin (AC-74) (Sigma-Aldrich: St Louis, USA) in a 1:80,000 dilution. Secondary antibodies goat anti-rabbit and goat anti-mouse were conjugated with horseradish peroxidase and used in 1:5,000 dilutions (Dako, Glostrup, Denmark).

### Co-immunoprecipitation

For Co-IP 5×10^6^ cells were lysed in 1x RIPA buffer (Cell Signaling), including protease inhibitor cocktail (Roche). 500 μg of each lysate were used according to the operating instructions of protein A/G PLUS-agarose protocol (Santa Cruz Biotechnology, Dallas, TX, USA). 500 μg of cellular protein dosed with 2 μg of anti-BATF3 antibody (3H1) (Abnova) were incubated for 1 hour at 4°C. Afterwards 20 μl of protein A/G-agarose were added and again incubated at 4°C overnight. After overnight incubation the samples were prepared and used for Western blot analysis. Western blots were analyzed for BATF3, JUN and Hsp70.

### Gene expression profiling

Gene expression profiling data were taken from data sets GSE 12453 [16] and GSE 14879 [15].

### Immunohistochemistry

Immunohistochemical stainings were performed on sections of formalin fixed and paraffin embedded HL cases (n = 12), DLBCL cases (n = 21), ALCL cases (n = 18) and murine tumor-infiltrated organs. Slides were stained with rabbit anti-mouse Bcl6 (EPR11410-43, Abcam, Cambridge, United Kingdom), PNA (Vector Laboratories, Burlingame, CA, USA) and rabbit anti-human BATF3 antibody (Abcam). For detection, the EnVision System-HRP (DAKO) was used.

### Mutation analysis of *BATF3*

DNA was isolated (DNeasy Blood & Tissue Kit, Qiagen, Hilden, Germany) from whole tissues slices of DLBCL cases (n = 21), ALCL cases (n = 18) and three HL cell lines (L-428, L1236, KM-H2) and the exons of *BATF3* were amplified via nested polymerase chain reaction (PCR) (Supplementary Table 2). Amplicons were compared with the reference sequence (NCBI, Gene ID: 55509) after Sanger sequencing.

### Ligation-mediated polymerase chain reaction (LM-PCR)

LM-PCR was performed as previously described [17, 18]. The strategy of this method is based on the selective amplification of genomic sequences adjacent of the proviral insertion site.

## SUPPLEMENTARY MATERIALS FIGURES AND TABLES



## Abbreviations

ALCL: anaplastic large cell lymphoma
DLBCL: diffuse large B-cell lymphoma
GEP: gene expression profiles
HRS: Hodgkin and Reed/Sternberg
HL: Hodgkin lymphoma
IRES: internal ribosomal entry site
MM: multiple myeloma
TCR: T cell receptor
