# Supplementary material for: Ectopic expression of transcription factor BATF3 induces B-cell lymphomas in a murine B-cell transplantation model
Source: Oncotarget. 2018 Mar 23;9(22):15942–51. doi: 10.18632/oncotarget.24639 (PMC5882309; doi:10.18632/oncotarget.24639)
Supplement: Supplementary file 1 [file oncotarget-09-15942-s001.pdf]

Ectopic expression of transcription factor BATF3 induces B-cell lymphomas in a murine B-cell transplantation model

SUPPLEMENTARY MATERIALS

REFERENCES

1. Schambach A, Wodrich H, Hildinger M, Bohne J, Kräusslich HG, Baum C. Context dependence of different modules for posttranscriptional enhancement of gene expression from retroviral vectors. Mol Ther J Am Soc Gene Ther. 2000; 2: 435–45. https://doi.org/10.1006/mthe.2000.0191.
2. Weber K, Bartsch U, Stocking C, Fehse B. A multicolor panel of novel lentiviral "gene ontology" (LeGO) vectors for functional gene analysis. Mol Ther J Am Soc Gene Ther. 2008; 16: 698–706. https://doi.org/10.1038/mt.2008.6.

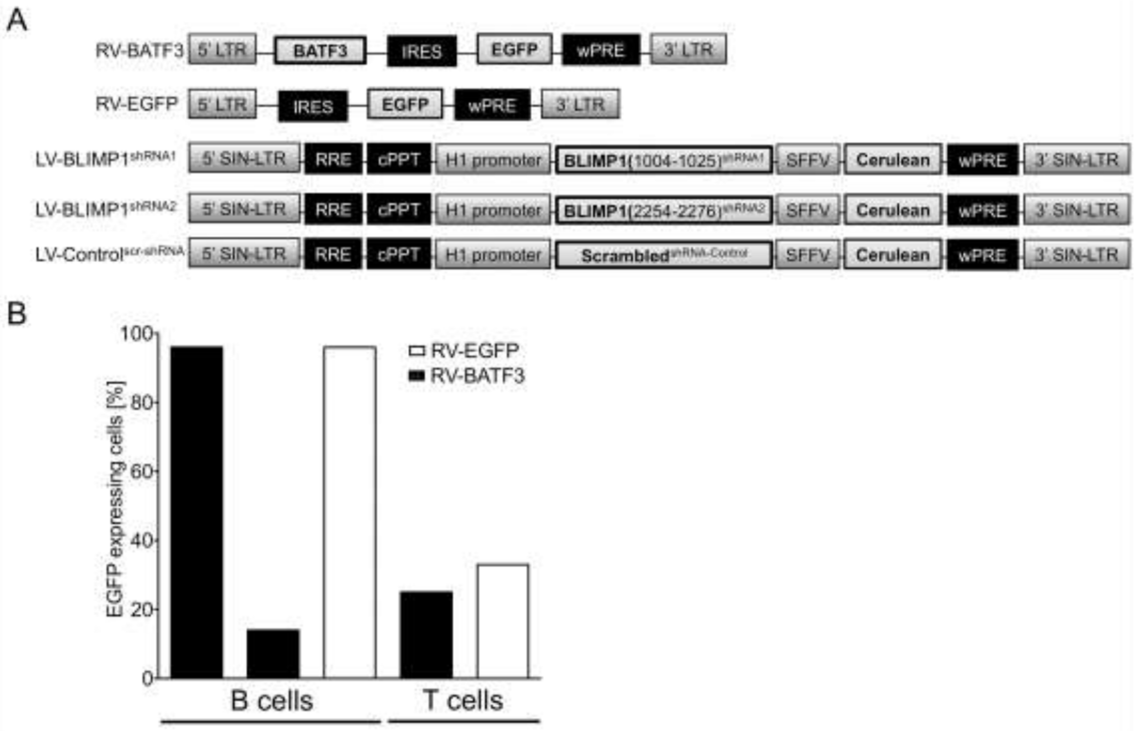

Supplementary Figure 1: Design of the retroviral vectors used in this study and transduction efficiencies of primary murine T and B cells. (A) The cDNA of the human *BATF3* gene was cloned into the gammaretroviral vector MP91-EGFP [1]. Sequences of *BLIMP1*-specific shRNAs and a non-specific scrambled control-shRNA were cloned into the lentiviral vector LeGO-Cer [2]. Targeted *BLIMP1*-sequences of shRNA1 and shRNA2 depicted in brackets. RV, gammaretroviral vector; LV, lentiviral vector; EGFP, enhanced green fluorescent protein; LTR, long terminal repeat; SIN-LTR, self-inactivating long terminal repeat; IRES, internal ribosomal entry site; RRE, Rev response element; cPPT, central polypurine tract; SFFV, spleen focus-forming virus; wPRE, woodchuck hepatitis virus posttranscriptional regulatory element. **(B)** Gene marking of transplanted mature T and B cells after retroviral transduction with RV-Batf3 or control-gene encoding vector RV-EGFP. For B cells a highly *BATF3*-transduced and a low *BATF3*-transduced transplant was prepared.

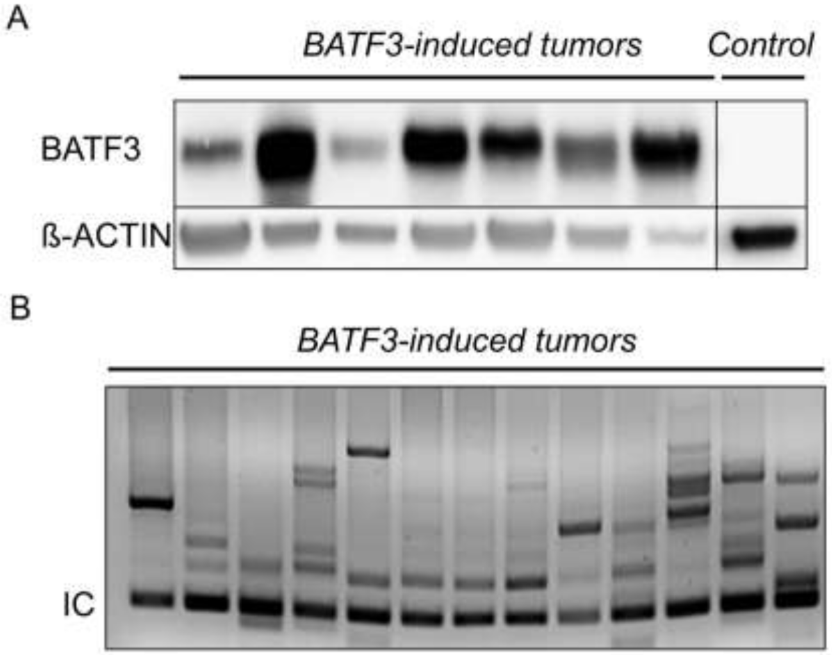

Supplementary Figure 2: BATF3 expression induced oligoclonal B-cell tumors. (A) Western blot of several BATF3-induced murine lymphomas demonstrated variable BATF3-expression levels. *In vivo* expanded and sorted B cells from the EGFP-control cohort served as negative control. **(B)** Retroviral integration analyses of BATF3-induced tumor material via LM-PCR. Every band represents a different integration site in the mouse genome. IC, internal control from vector sequence.

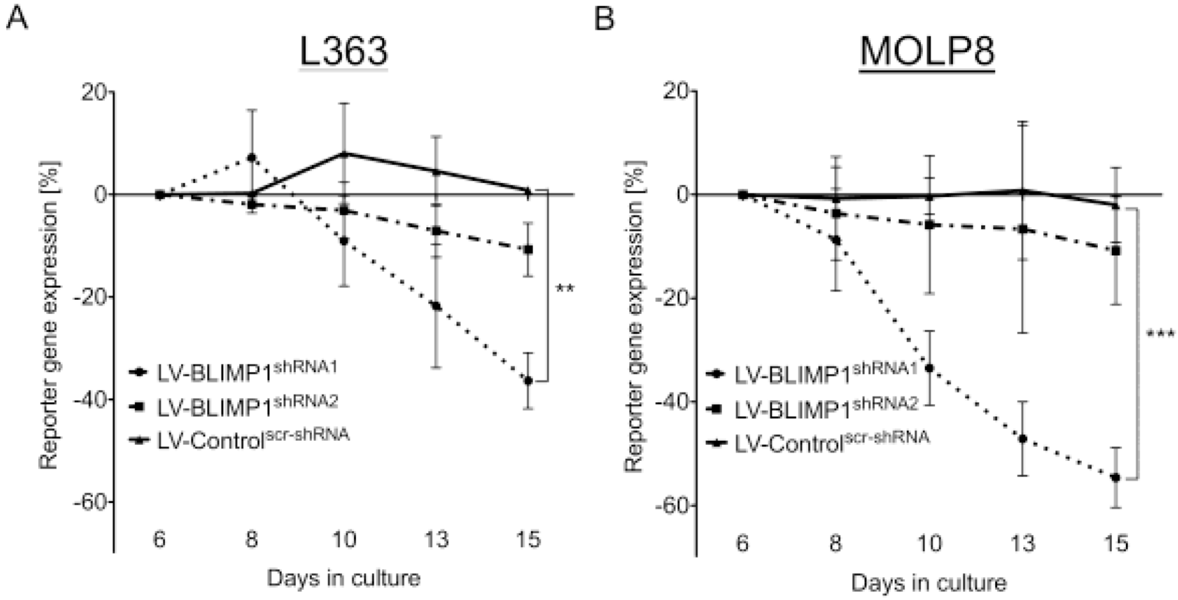

Supplementary Figure 3: Targeted knockdown of BLIMP1 in multiple myeloma cell lines MOLP8 and L363. Multiple myeloma cell lines L363 **(A)** and MOLP8 **(B)** were either transduced with two different BLIMP1-targeting shRNAs ($BLIMP1^{shRNA1}$ and $BLIMP1^{shRNA2}$) or a non-specific, scrambled shRNA as control ($Control^{scr\text{-}shRNA}$). After two weeks of culture, BLIMP1-specific knockdown with shRNA1 resulted in a dramatically impaired cell expansion of reporter gene expressing cells in both cell lines. Data from three independent experiments are shown. Error bars represent standard deviation. Statistical significance was established with a paired *t* test. **, $P < 0.01$, ***, $P < 0.0001$

Supplementary Table 1: Phenotype of transplanted cells

Group	CD19/B220 double positive	GL7/Fas double positive	CXCR4	CXCR5	IgM	IgD	IgG	Igλ	Igκ	CD86	CD83
RV-EGFP	99.2	99.8	21.0	82.2	88.9	74.3	10.2	17.6	81.0	97.5	52.0
RV-BATF3	98.7	98.9	18.9	80.1	92.8	72.1	11.8	21.1	75.4	96.1	57.7

Supplementary Table 2: Primers for amplification of the coding sequence of *BATF3*

Primer	Sequence 5′ → 3′
EX1 FW1	TGC GGC ACG AGG ATG CC
EX1 FW2	TAG GCA GCC CCA CGG GC
EX1 RV1	CTG GAG TTC CGT GGT GGT GA
EX1 RV2	GGA GAC AAG CAG AGG TAG GG
EX2 FW1	GGT GCT GTC TAC TGC AAA GC
EX2 FW2	AGA AAA GGG TAA GGC GAG G
EX2 RV1	CTA ATT TCT GCC AGG TCC TTC C
EX2 RV2	CTG AGT GCT TCT CAT GGT CA
EX3 FW1	GCT TTC ATG GGC AAG AGG TG
EX3 FW2	GAG GAA GGG AAC GCT GC
EX3 RV1	CTC AGC CCG ACA TCC AAC A
EX3 RV2	AGA TCC AGC ATG GAG GCC A

EX, exon; FW, forward primer; RV, reverse primer.
